# Real-world effectiveness of CDK 4/6 inhibitors in estrogen-positive metastatic breast cancer

**DOI:** 10.1038/s44276-024-00070-w

**Published:** 2024-06-20

**Authors:** Mathilde Louise Gehrchen, Tobias Berg, Rasmus Garly, Maj-Britt Jensen, Saskia Eßer-Naumann, Jeanette Dupont Rønlev, Hanne Melgaard Nielsen, Ann Knoop, Iben Kümler

**Affiliations:** 1 Danish Breast Cancer Group, Department of Oncology, Copenhagen University Hospital, Rigshospitalet, Copenhagen, Denmark; 2 Department of Oncology, Copenhagen University Hospital, Rigshospitalet, Copenhagen, Denmark; 3 Department of Oncology, Næstved Hospital, Næstved, Denmark; 4 Department of Oncology, University Hospital of Odense, Odense, Denmark; 5 Department of Oncology, Aarhus University Hospital, Aarhus, Denmark; 6 Department of Oncology, Herlev and Gentofte University Hospital, Herlev, Denmark; 7 Department of Clinical Medicine, Faculty of Health and Medical Sciences, University of Copenhagen, Copenhagen, Denmark

## Abstract

**Background:**

Initial treatment for advanced ER-positive/HER2-negative breast cancer involves a CDK 4/6 inhibitor (CDK 4/6i). Recent overall survival (OS) analyses led the Danish Medical Council to exclude palbociclib as preferred option. This study aimed to evaluate the real-world effectiveness of abemaciclib, palbociclib, and ribociclib in a Danish context. Additionally, to compare the inhibitors to identify potential endpoint differences.

**Material and methods:**

Patients undergoing first or second line CDK 4/6i treatments from January 1st, 2017, until December 31st, 2021 were included. The primary endpoint was progression free survival (PFS).

**Results:**

Among 2069 Danish patients, 1554 received first line treatment, 515 received second line treatment. In first line, abemaciclib’s median PFS was unreached; palbociclib had a median PFS of 32.0 months (95% CI: 28.9–35.3); ribociclib 42.4 months (95% CI: 35.1–52.9). First-line median OS was 37.8 months (95% CI: 32.5–NA); 49.7 months (95% CI: 44.7–54.1); and 54.4 months (95% CI: 47.9–NA) for abemaciclib, palbociclib and ribociclib, respectively. No significant differences in OS were observed, nor in PFS in second line.

**Conclusion:**

This study confirms first-line CDK 4/6i effectiveness, with abemaciclib and ribociclib showing prolonged PFS vs. palbociclib. This study could not confirm a ranking of the three CDK 4/6i.

## Background

Approximately 73% of all breast cancers (BC) are estrogen receptor (ER) positive [1]. For patients with metastatic ER-positive and human epidermal growth factor receptor 2 (HER2) negative BC the 5-year relative survival rate is 34% [2, 3]. International guidelines recommend treating this type of BC with a CDK 4/6 inhibitor (CDK 4/6i) in combination with endocrine therapy (ET), i.e. an aromatase inhibitor (AI) or fulvestrant, as first-line treatment [4]. CDK 4/6i halt the cell cycle in tumor cells with excelled CDK 4/6 activity [5]. Moreover, recent findings suggest that some mutations causing endocrine resistance still respond to CDK 4/6i treatment [6]. The CDK 4/6i treatment was introduced in Denmark in 2017 with the approval of palbociclib, followed by ribociclib and abemaciclib in 2018 and 2020, respectively.

Newly published overall survival (OS) analyses from randomized trials show a notably increased OS for abemaciclib and ribociclib, but not for palbociclib [7–9]. Consequently, as of May 24th, 2023, the Danish Medical Council revised its guidelines, now recommending abemaciclib and ribociclib as the primary choices for advanced or metastatic ER-positive/HER2-negative BC [10]. Internationally, ESMO-MCBS scores the three inhibitors differently however both ASCO and ESO-ESMO guidelines still recommend all three CDK 4/6i equally [11, 12]. Thus, there is no clear ranking of the inhibitors as of yet. Inhibitor selection based on effectiveness is now a debated topic and further research is necessary to determine whether there in fact should be a clear ranking [13]. Thus, in the aftermath of the randomized trials, gathering real-world data is crucial in order to ascertain the effectiveness of the CDK 4/6i in a general population. The objective of this study was to investigate the use of all three CDK 4/6i employed in Denmark. The goal was not only to compare their effectiveness with that observed in randomized trials but also draw comparisons among the inhibitors themselves. To the best of our knowledge, comprehensive real-world data that compares all three inhibitors is yet to be published.

## Material and methods

### Study design and setting

This study adheres to STROBE guidelines for observational studies [14]. All patients initiating CDK 4/6i treatment in first or second line between January 1st, 2017, until December 31st, 2021, were included in this retrospective cohort study. Patients were included under the assumption that only ER-positive/HER2-negative patients are treated with a CDK 4/6i. Patients were eligible in first line without prior treatment for metastatic disease and in second line without CDK 4/6i treatment in first line.

Data collection utilized the Danish Breast Cancer Group (DBCG) database for recurrent BC, encompassing nationwide data from oncology departments. The database is updated continuously. Available variables included: Date of diagnosis, date of recurrence, number of metastases, type of metastases, death date, start and end of treatment, type of treatment, reason for discontinuation of treatment, and follow-up data. Adjuvant endocrine data was obtained from the DBCG adjuvant database. Endocrine sensitivity followed ESMO guidelines [12]. If adjuvant data was missing, sensitivity was assumed if ≥6 years had passed from primary diagnosis until recurrence. All patient information was updated until April 1st, 2023.

### Outcomes

The primary outcome was progression free survival (PFS) and secondary outcomes were overall survival (OS) and time on treatment (ToT). PFS was defined as date of metastatic disease to progression or death in first line; first progression to next progression or death in second line. Disease progression was verified by the individual clinicians at the oncology departments and included clinical, radiological, and biochemical measures. OS was defined as metastatic disease date to any cause of death in first line; first progression to any cause of death in second line. ToT was defined as CDK 4/6i treatment start until treatment stop, considering death as a competing event and censoring patients still on treatment by the end of follow-up. Switch between different CDK 4/6i within the treatment line was not accounted for.

### Statistical analysis

Categorical variables were described as numbers and proportions and compared using the chi square test or Fischer’s exact test. Numeric age was described by median and range, was non-normally distributed, and compared using the Kruskal–Wallis test. The Kaplan–Meier method was used to estimate PFS and OS with 95% confidence intervals (CI). The log-rank test was used to compare groups. The Cox proportional hazards regression model was applied to estimate the hazard ratio (HR) with corresponding 95% CI of PFS for patients with CDK 4/6i in first line. Variables with a *p* < 0.1 in the univariable model were included for multivariable analysis. No imputation method was used for multivariable analysis. The proportional hazard assumption was tested by Schoenfeld residuals. A cumulative incidence function was used to describe ToT, with death as a competing event. Potential median follow-up was estimated by use of a reverse Kaplan-Meier method [15]. A *p*-value < 0.05 was considered statistically significant. All data analyses were conducted in R version 4.2.2.

## Results

### Study population

**Fig. 1:**
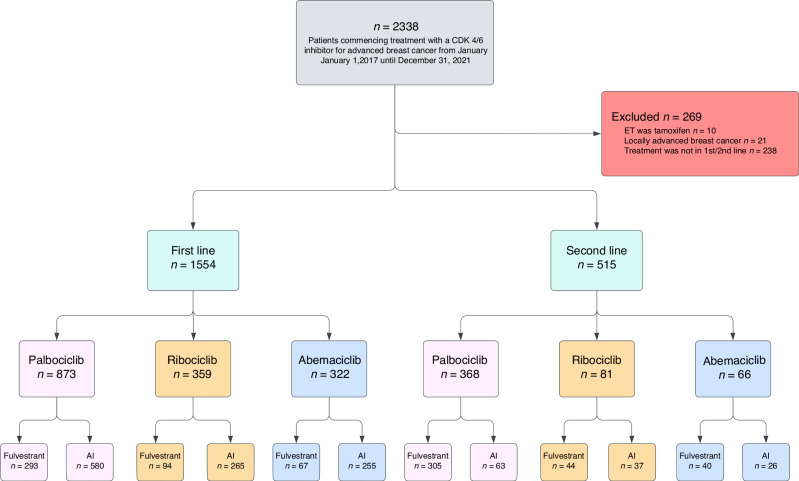
Flowchart of the study cohort. Patients were divided in subgroups based on line of treatment, type of CDK4/6i, and type of endocrine backbone.

During 2017–2021, 2338 ER-positive/HER2-negative BC patients commenced treatment with a CDK4/6i. We excluded 269 patients, for non-first or -second line use (*n* = 238), locally advanced only (*n* = 21) and tamoxifen as endocrine backbone (*n* = 10). The cohort came to consist of 2069 patients with metastatic BC of which 1554 received treatment in first line and 515 received treatment in second line (Fig. 1).

#### CDK 4/6i treatment in first line

In first line, 873 patients received palbociclib, 359 received ribociclib, and 322 patients received abemaciclib. The median age was 69.3 [range 22.7, 92.8] and near half aged ≥70. For first-line patients, 53.9% (*n* = 837) had visceral disease and 26.2% (*n* = 407) had bone-only disease. 1131 patients (72.8%) had recurrent metastatic BC; 423 (27.2%) had primary metastatic BC. 1100 patients (70.8%) received an AI as endocrine backbone whilst 454 (29.2%) received fulvestrant (Table 1).

**Table 1:** Baseline characteristics for patients receiving CDK 4/6i treatment in first line.

	Abemaciclib (*n* = 322)	Palbociclib (*n* = 873)	Ribociclib (*n* = 359)	Overall (*n* = 1554)	*P*-value
**Age**					
Median [range]	67.7 [32.0, 87.4]	69.7 [27.6, 92.8]	69.0 [22.7, 88.6]	69.3 [22.7, 92.8]	0.02
**Age**					
<40	8 (2.5%)	11 (1.3%)	8 (2.2%)	27 (1.7%)	0.01
40–49	38 (11.8%)	69 (7.9%)	22 (6.1%)	129 (8.3%)	
50–59	71 (22.0%)	142 (16.3%)	65 (18.1%)	278 (17.9%)	
60–69	61 (18.9%)	224 (25.7%)	93 (25.9%)	378 (24.3%)	
≥70	144 (44.7%)	427 (48.9%)	171 (47.6%)	742 (47.7%)	
**Year of metastatic disease**					
2017	0 (0%)	219 (25.1%)	9 (2.5%)	228 (14.7%)	<0.001
2018	0 (0%)	230 (26.3%)	107 (29.8%)	337 (21.7%)	
2019	5 (1.6%)	167 (19.1%)	149 (41.5%)	321 (20.7%)	
2020	110 (34.2%)	174 (19.9%)	81 (22.6%)	365 (23.5%)	
2021	207 (64.3%)	83 (9.5%)	13 (3.6%)	303 (19.5%)	
**No. of metastatic sites**					
1	136 (42.2%)	372 (42.6%)	164 (45.7%)	672 (43.2%)	0.64
2	93 (28.9%)	241 (27.6%)	86 (24.0%)	420 (27.0%)	
≥3	93 (28.9%)	260 (29.8%)	109 (30.4%)	462 (29.7%)	
**Sites of metastases**					
Non-visceral	79 (24.5%)	166 (19.0%)	65 (18.1%)	310 (19.9%)	0.047
Visceral^a^	164 (50.9%)	489 (56.0%)	184 (51.3%)	837 (53.9%)	
Bone-only	79 (24.5%)	218 (25.0%)	110 (30.6%)	407 (26.2%)	
**Disease presentation**					
Recurrent metastatic	217 (67.4%)	648 (74.2%)	266 (74.1%)	1131 (72.8%)	0.051
Primary metastatic	105 (32.6%)	225 (25.8%)	93 (25.9%)	423 (27.2%)	
**Endocrine backbone**					
AI^b^	255 (79.2%)	580 (66.4%)	265 (73.8%)	1100 (70.8%)	<0.001
Fulvestrant	67 (20.8%)	293 (33.6%)	94 (26.2%)	454 (29.2%)	

^a^Liver, lungs, CNS, abdominal carcinosis, and/or ovaries.

^b^Aromatase inhibitor.

CDK 4/6i distribution differed significantly over the study period, reflecting drug availability in the Danish market. The group receiving abemaciclib was younger compared to the palbociclib and ribociclib groups. The abemaciclib group also had more non-visceral metastases (24.5% vs. 19.0% for palbociclib and 18.1% for ribociclib) and more AI-based endocrine backbones (79.2% vs. 66.4% for palbociclib and 73.8% for ribociclib). The palbociclib group had more visceral metastases (56% vs. 50.9% for abemaciclib and 51.3% for ribociclib). The ribociclib group had more bone-only disease (30.6% vs. 24.5% for abemaciclib and 25% for palbociclib) (Table 1).

#### CDK 4/6i treatment in second line

In second line, 368 patients received palbociclib, 81 received ribociclib, and 66 received abemaciclib. The median age was 68.2 [range 26.6, 89.7]. Of these, 68.9% (*n* = 355) had visceral disease and 13.4% (*n* = 69) had bone-only disease. 389 patients (75.5%) received fulvestrant as endocrine backbone and 126 (24.5%) received an AI. The abemaciclib group had more recurrent metastatic BC (90.9% vs. 77.7% for palbociclib). The palbociclib group had significantly fewer AI-based endocrine backbones (17.1% vs. 39.4% for abemaciclib and 45.7% for ribociclib) (Supplementary Table 1). Compared to first line, second line had more fulvestrant-based endocrine backbones (75.5% vs. 29.2%) and more visceral metastases (68.9% vs. 53.9%).

### Survival analysis

#### Progression free survival

**Fig. 2:**
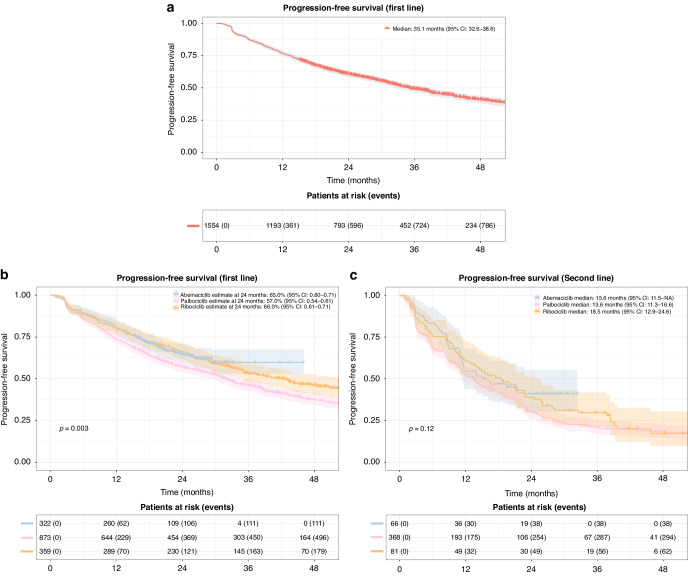
Progression-free survival (PFS). **a** Progression-free survival in patients receiving CDK 4/6i treatment in first line. Shaded areas represent confidence intervals. **b** Progression-free survival in patients receiving CDK 4/6i treatment in first line grouped by CDK 4/6i type. Shaded areas represent confidence intervals. **c** Progression-free survival in patients receiving CDK 4/6i treatment in second line grouped by CDK 4/6i type. Shaded areas represent confidence intervals.

Among 2069 patients, 1211 (58.5%) experienced an event. First-line CDK 4/6i recipients had a median PFS of 35.1 months (95% CI: 32.6–38.6) (Fig. 2a). Divided by CDK 4/6i type, first-line median PFS for palbociclib was 32.0 months (95% CI: 28.9–35.3); for ribociclib 42.4 months (95% CI: 35.1–52.9); for abemaciclib not reached, however the survival probability at the 24-month mark was 65.0% (95% CI: 0.60–0.71), and for palbociclib and ribociclib: 57% (95% CI: 0.54–0.61) and 66% (95% CI: 0.61–0.71), respectively. There was a significantly prolonged PFS for abemaciclib (unadjusted HR 0.77, 95% CI: 0.62–0.94, *p* = 0.01) and ribociclib (unadjusted HR 0.78, 95% CI: 0.66–0.93, *p* = 0.004) compared to palbociclib (Fig. 2b). Second-line median PFS for abemaciclib was 15.6 months (95% CI: 11.5–NA); for palbociclib 13.6 months (95% CI: 11.3–16.6); for ribociclib 18.5 months (95% CI: 12.9–24.6). There was no significant difference when comparing abemaciclib and ribociclib to palbociclib (Fig. 2c).

Endocrine sensitivity had a significant influence on PFS in first-line patients (Supplementary Fig. 1a). The endocrine sensitive patients had a median PFS of 40.2 months (95% CI: 37.1–45.2) whilst endocrine resistant patients, including both primary and secondary resistance, had a median PFS of 21.6 months (95% CI: 19.1–26.0). We found no statistically significant difference in PFS for second-line patients (Supplementary Fig. 1b). Our multivariable cox proportional hazards regression model showed that patients treated with both abemaciclib and ribociclib fared better than patients treated with palbociclib, HR 0.74 (95% CI: 0.60–0.90), *p* = 0.005 for abemaciclib and HR 0.80 (95% CI: 0.68–0.96), *p* = 0.01 for ribociclib, when adjusting for age, sites of metastases, endocrine backbone, and endocrine sensitivity (Supplementary Table 2).

#### Overall survival

**Fig. 3:**
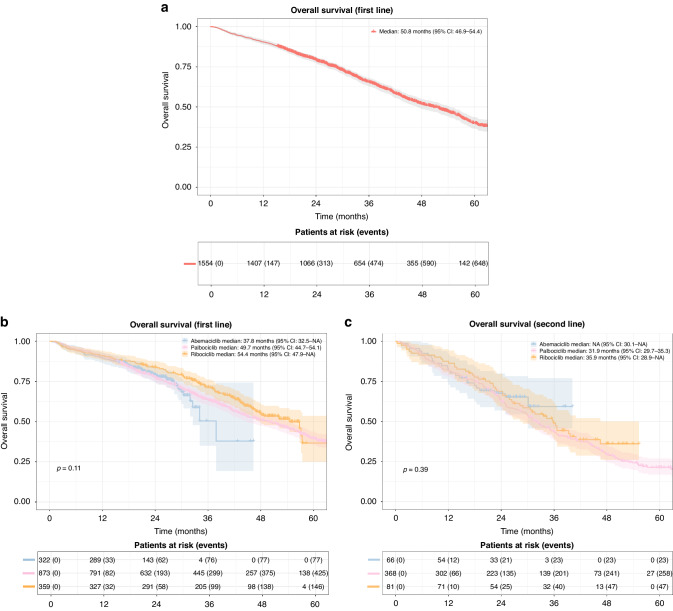
Overall Survival (OS). **a** Overall survival in patients receiving CDK 4/6i treatment in first line. Shaded areas represent confidence intervals. **b** Overall survival in patients receiving CDK 4/6i treatment in first line grouped by CDK 4/6i type. Shaded areas represent confidence intervals. **c** Overall survival in patients receiving CDK 4/6i treatment in second line grouped by CDK 4/6i type. Shaded areas represent confidence intervals.

Among 2069 patients, 994 (48%) died before data cut-off. First-line CDK 4/6i recipients had a median OS of 50.8 months (95% CI: 46.9–54.4) (Fig. 3a). Divided by CDK 4/6i type, first-line median OS for abemaciclib was 37.8 months (95% CI: 32.5–NA); for palbociclib 49.7 months (95% CI: 44.7–54.1); for ribociclib 54.4 months (95% CI: 47.9–NA). There was no significant difference in OS when comparing the three groups (Fig. 3b). Second-line median OS for abemaciclib was not reached; for palbociclib 31.9 months (95% CI: 29.7–35.3); for ribociclib 36.0 months (95% CI: 28.9–NA). There was no significant difference in OS when comparing the three treatment groups in second line (Fig. 3c).

### Treatment characteristics

**Fig. 4:**
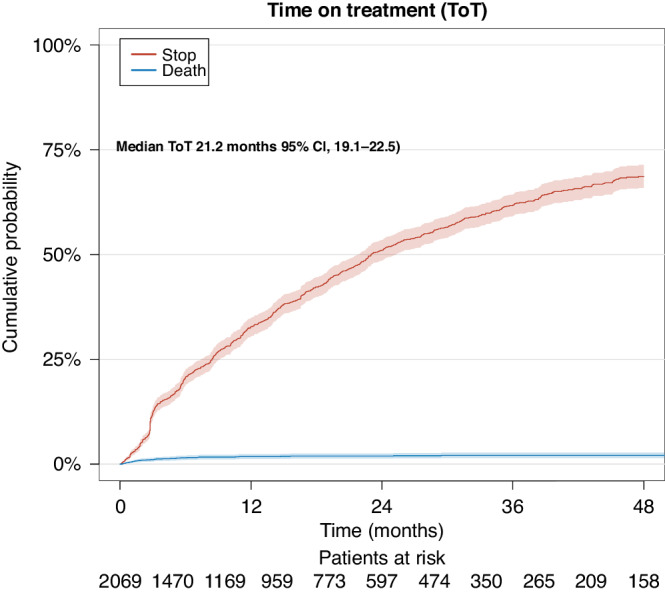
Competing risk analysis of time on treatment (ToT). The blue curve represents patients who discontinue CDK 4/6i treatment due to death of any cause. The red curve represents patients who discontinue CDK 4/6i treatment due to other reasons, i.e. progression, toxicities, and change in treatment etc.

Median ToT for the entire cohort was 21.2 months (95% CI: 19.1–22.5). Cumulative probability of death on CDK 4/6i treatment was minimal (Fig. 4). Overall median follow-up was 45.7 months (95% CI: 44.3–47.5) however, follow-up varied by treatment group: abemaciclib had notably shorter median potential follow-up than palbociclib and ribociclib in both lines. First-line abemaciclib: 24.8 months (95% CI: 24.1–25.8); palbociclib: 55.1 (95% CI: 52.1–58.0); ribociclib: 47.9 (95% CI: 45.8–49.7). The results were similar for second line.

In first line, 172 patients switched CDK 4/6i types. Cross-over increased palbociclib as second or third choice, while abemaciclib, initially largest, became smallest due to in-line changes. The same pattern applied to second-line treatment (Supplementary Fig. 2a, b). Palbociclib and ribociclib were mostly discontinued due to progression (50.3% and 40.5%, respectively) while abemaciclib was discontinued primarily due to toxicities (42.3%) (Supplementary Table 3).

### Endocrine sensitivity

#### CDK 4/6i treatment in first line

Regarding endocrine sensitivity in first line treatment, AI-based endocrine backbone was dominated by endocrine sensitive patients in all treatment groups. For fulvestrant-based endocrine backbone abemaciclib patients, fewer were endocrine sensitive (40.3% vs. 60.4% for palbociclib and 54.3% for ribociclib) and more patients were secondary resistant (47.8% vs. 28% for palbociclib and 36.2% for ribociclib) (Supplementary Table 4a).

#### CDK 4/6i treatment in second line

Similar to first line, AI-based backbones were mostly hormone sensitive. Secondary resistance was more common than in first line: abemaciclib and AI had 9.8% secondary resistance in first line compared to 42.3% in second line. The majority of patients treated with fulvestrant were secondary resistant. There were no significant differences when comparing CDK 4/6i groups (Supplementary Table 4b).

## Discussion

### Key results

This study assessed the effectiveness of CDK 4/6i within the Danish population using retrospective data. Abemaciclib and ribociclib in first line treatment showed a prolonged PFS compared to palbociclib, remaining significant in an adjusted multivariable model. Age, sites of metastases, endocrine backbone, and endocrine sensitivity influenced first-line PFS. For OS, there were no statistically significant differences between the inhibitors, despite ribociclib having a longer median OS in both first and second-line patients.

### Strengths and limitations

In terms of strengths and limitations, several aspects deserve consideration. First, Table 1 reveals the increasing use of abemaciclib. However, it is noteworthy that the palbociclib group stands larger and with a longer follow-up duration attributed to its earlier approval compared to abemaciclib. Despite this, efforts ensured a follow-up of at least 12 months for all patients, maintaining a relatively substantial abemaciclib sample size. Limited follow-up for abemaciclib, and ribociclib in second line, hindered accurate PFS and OS estimates as well as confidence intervals. Due to the time-varying introductions of the CDK 4/6i to the market, there is a clear discrepancy between the three treatment groups in this study regarding follow-up. In efforts to make comparisons between the inhibitors easier we were able to calculate the survival probability at 24 months for all three inhibitors. Secondly, due to the novelty of CDK 4/6i treatment some patients missed initial first-line treatment due to their time of disease recurrence, influencing second-line ratios with seemingly fewer patients towards 2021. This suggests that the treatment has become more established as first-line treatment for metastatic BC.

Furthermore, there were 78 patients with data missing on adjuvant endocrine therapy that were deemed endocrine sensitive due to 6 or more years between primary diagnosis and recurrence. Some of these cases could be misclassified although we argue that it has negligible result impact. Finally, the choice of CDK 4/6i is influenced by the individual clinician’s considerations. It is also the individual clinician who verifies progression, thus there is no way of knowing if there exist cases where the patient has been deemed in progression due to solely clinical progression, i.e. not verified by scans. We argue that these cases are few since the clinicians follow national guidelines as a rule. In addition, regional differences in inhibitor choice introduce unaccounted selection bias, given the absence of guidelines recommending a certain inhibitor above the others during the study period.

The current study boasts several strengths in addition to acknowledging its limitations. It is founded on a population-based cohort of considerable size, minimizing the risk of type II errors, and holds real-world Danish applicability. The retrieval of up-to-date follow-up data further solidifies the integrity of this comprehensive cohort.

### Interpretation

#### Study population and treatment characteristics

As displayed in Supplementary Fig. 2, the distribution of patients switching CDK 4/6i within first-line treatment varied across the treatment groups. The introduction of palbociclib in Denmark in 2017 as the sole option might explain its lower rate of patients switching. Conversely, the abemaciclib group had a larger number of patients opting for a second choice of CDK 4/6i given the availability of both palbociclib and ribociclib. Additionally, the abemaciclib group had more than double the number of treatment discontinuations due to toxicities than both palbociclib and ribociclib (Supplementary Table 3). Many patients likely transitioned to a different CDK 4/6i due to distinct toxicity profiles. Abemaciclib especially has toxicities related to the gastro-intestinal system whereas palbociclib and ribociclib have toxicities related to myelosuppression [5, 16]. Our findings support previous research which has shown that abemaciclib prompts more toxicity related treatment discontinuations [5]. Thus, it seems that palbociclib and ribociclib provoke adverse events that are primarily abnormalities shown on lab results without necessarily affecting the quality of life whereas for abemaciclib, the gastro-intestinal toxicities can be highly intolerable.

Lastly, the patients that received palbociclib treatment in first line had more visceral metastases and the multivariable analysis confirmed the significance of sites of metastases on PFS. Since palbociclib was the first CDK 4/6i type to be used in Denmark, it is likely that clinicians were stricter with the use of it and that it was chosen for patients with more serious conditions whereas the use is more loose today. Even though our results showed a significant effect of sites of metastases on PFS, we adjusted for this in our multivariable analysis and type of CDK 4/6i was still significant, which reduces the potential confounder.

#### Progression free survival compared to randomized trials

Our study revealed longer PFS results compared to randomized phase III studies. The PALOMA-2 study found a median PFS for palbociclib and letrozole of 27.6 months (95% CI: 22.4–30.3) [17] whereas our study found a median PFS for palbociclib with AI or fulvestrant of 32.0 months (95% CI: 28.9–35.3) in first-line patients. Unlike PALOMA-2, our study included both post- and premenopausal women, varied endocrine backbones (letrozole, anastrozole, exemestane, fulvestrant), and real-world patients. The real-world context was anticipated to yield a shorter PFS, especially since the current study included more palbociclib patients with visceral metastases than PALOMA-2 (71.5% vs. 48.2%). Additionally, our multivariable model noted higher progression risk with anastrozole and fulvestrant compared to letrozole. However, previous research has found fulvestrant to be equally beneficial to an AI and international guidelines for first-line treatment recommend both as options [10, 18]. Nevertheless, our longer median PFS compared with PALOMA-2 hints at the promise of real-world outcomes, although one should take note of the overlapping confidence intervals between PALOMA-2 and our study and the fall-pits of cross-study comparison.

The MONALEESA-2 study, a phase III randomized study, found a median PFS for ribociclib and letrozole of 26.4 months (95% CI: 23.0–30.3) [19] where our results showed a median PFS of 42.4 (95% CI: 35.1–52.9) which was significantly higher. The differences were similar as those described between the current study and the PALOMA-2 study. Looking at abemaciclib, the MONARCH-3 study showed a median PFS of 28.18 months [20], whereas our study found a survival probability of 65.0% at 24 months. This suggests a potential median PFS aligning with MONARCH-3, although it is necessary to extend the follow-up period for abemaciclib in the current study to make a conclusive analysis. These findings emphasize the favorable real-world effectiveness of CDK 4/6i treatment regarding PFS.

Comparing abemaciclib, palbociclib, and ribociclib in the current study, ribociclib had a significantly longer median PFS than palbociclib. However, several baseline differences existed, for instance the palbociclib group having more visceral metastases. Despite recognizing the influence of the sites of metastases on PFS, the multivariable model continued to show a significant difference in PFS between the inhibitors when adjusting for factors like metastatic sites. Furthermore, when comparing the survival probability at 24 months in first-line patients, the difference between treatment groups bordered the significance level with a lower survival probability for palbociclib. A possible explanation for the shorter PFS for palbociclib could be that evaluation scans were performed more frequently, i.e. every 3 months. Because ER-positive breast cancer evolves more slowly the clinicians have begun to evaluate every 4 or 6 months after the first year, if the patient experienced no symptoms. This means that for ribociclib and abemaciclib progression is potentially detected later, causing potential bias.

#### Progression free survival compared to real world studies

In recent years, a handful of studies have established the benefits of CDK 4/6i in a real-world population compared to AI monotherapy [21, 22]. A small retrospective study found 140 patients receiving CDK 4/6i treatment between 2014 and 2019 [23]. They had a median age of 65 similar to our study population and similarly a majority of recurrent breast cancer with visceral metastases. Furthermore, discontinuation of treatment was primarily due to progression which correlates to our findings. They found a median PFS of 6.3 months (95% CI: 5.1–7.4) which was significantly lower than ours. It should be noted that the majority of the patients in the study by West et al. had received treatment for metastatic breast cancer before treatment with CDK 4/6i. Thus, they did not receive CDK 4/6i treatment in first line, which makes the comparison to the current study problematic.

To the best of our knowledge, studies have so far established the benefits of CDK 4/6i, but the large part of the studies include primarily palbociclib and thus far, we have found no real-world studies that compare the three CDK 4/6i to each other.

#### Overall survival compared to randomized trials

We found a median OS for patients that received any CDK 4/6i in first line of 50.8 months (95% CI: 46.9–54.4). Deluche et al. investigated real-world outcomes for ER-positive/HER2-negative metastatic BC, reporting a median OS of 43.3 months (95% CI: 42.5–44.5) for 22000 patients treated between 2008 to 2016, predating CDK 4/6i use [3]. The current study’s real-world data, encompassing 1554 receiving a CDK 4/6i as first-line treatment for metastatic BC, suggests significant treatment benefits.

The current study’s OS outcomes stratified by CDK 4/6i type differed from randomized trials. In the MONARCH-3 interim OS analysis, Goetz et al. reported a median OS of 67.1 months (confidence intervals not published yet) with 70.2 months median follow-up for abemaciclib and a non-steroidal AI [7]. The current study found 37.8 months median OS (32.5–NA) with 24.8 months median follow-up, a high censoring rate preventing upper CI estimation for abemaciclib. For palbociclib, with 55.1 months median follow-up, our median OS was 48.7 months (95% CI: 44.7–54.1), similar to PALOMA-2’s 53.9 months (95% CI: 49.8–60.8) for palbociclib and letrozole [9]. For ribociclib we were able to obtain a median follow-up of 47.9 months, which gave a median OS of 54.4 months (47.9-NA). MONALEESA-2 reported a median OS of 63.9 months (95% CI: 52.4–71.0) with a longer follow-up of 6.6 years [8]. The limited CI in the current study hinders precise cross-study comparison.

No significant difference was observed in OS between the treatment groups. Extending follow-up, especially for abemaciclib, is essential for more accurate OS estimates. PALOMA-2 reported no significant difference in OS between the palbociclib group and the placebo group while MONARCH-3 and MONALEESA-2 reported benefit with, respectively, abemaciclib and ribociclib compared to placebo groups [7–9]. Further investigations are needed to validate if palbociclib’s OS benefit is in fact inferior to abemaciclib and ribociclib. Although the OS for ribociclib was notably prolonged compared to palbociclib in the current study, the difference is not statistically significant, likely due to the duration of follow-up. Extended follow-up would clarify the significance more accurately. Additionally, information on subsequent treatment after progression on CDK 4/6i treatment could provide valuable knowledge in the context of overall survival.

#### Endocrine sensitivity

The current study revealed statistically significant PFS improvement in first-line endocrine sensitive patients vs. first-line endocrine resistant patients (Supplementary Fig. 1a). The influence of endocrine sensitivity on PFS was significant in both the univariate end the multivariate models (Supplementary Table 2). This aligns with established research indicating that long term ET induces acquired endocrine resistance [24].

PALOMA-3 investigated the efficacy of palbociclib and fulvestrant in patients who had relapsed or progressed during prior ET [25]. Per ESMO guidelines, these patients would be categorized as endocrine resistant [12]. Their PFS of 9.2 months (95% CI: 7.5 to not estimable) was shorter than results from PALOMA-2 based on mainly endocrine sensitive patients. The current study is in accordance with those findings, showing shorter PFS in endocrine resistant patients as well as in second-line patients compared with first-line patients, indicating the need to address endocrine resistance in metastatic BC. Similarly, MONARCH-2 found a median OS of 16.4 months in endocrine resistant patients – significantly shorter than MONARCH-3 results in endocrine sensitive patients [26].

In general, fulvestrant was used more commonly as endocrine backbone in the palbociclib groups in first and second line compared to ribociclib and abemaciclib (Table 1 and Supplementary Table 1). Palbociclib was the initial CDK 4/6i in Denmark and patients were likely treated with endocrine monotherapy before it, making fulvestrant the natural endocrine backbone choice for second-line patients. Alternatively, physician preference might drive backbone selection considering AI and fulvestrant are considered equally beneficial, as previously discussed [10, 18].

Furthermore, patients treated with first line CDK 4/6i differed significantly in endocrine sensitivity (Supplementary Table 4a). The palbociclib group had more endocrine sensitive patients who received fulvestrant. It is important to recognize that a significant difference in endocrine backbone between the treatment groups makes it difficult to correctly compare the subgroups of endocrine backbone regarding endocrine sensitivity.

## Conclusions

The current study is thus far the largest real-world study to confirm CDK 4/6i treatment benefits in metastatic BC. PFS was significantly prolonged for abemaciclib and ribociclib compared to palbociclib, maintaining a significance after adjusting for other influential variables. Ribociclib showed a longer median OS in both lines, however the difference was lacking statistical significance. The findings of the current study correlate with existing research that has found palbociclib to be inferior to the other CDK 4/6i, however it was not possible to fully establish a ranking order of the three drugs. Whether the worse outcome for patients receiving palbociclib in this study is caused by the drug itself or is due to other influences is not clear. Further investigation as well as expanding follow-up will enhance PFS and OS analysis accuracy, finally validating or dismissing tendencies revealed in this study.

## Supplementary information

Supplementary information

## Data Availability

All data are stored in the DBCG database. The dataset can be made available to qualified researchers through application to the Danish Breast Cancer Group. Please contact dbcg.rigshospitaletegionh.dk.

## References

[1] Howlader N, Altekruse SF, Li CI, Chen VW, Clarke CA, Ries LAG, et al. US incidence of breast cancer subtypes defined by joint hormone receptor and HER2 status. J Natl Cancer Inst. 2014;106:dju055. 24777111 10.1093/jnci/dju055 PMC4580552

[2] SEER*Explorer Application. 2023. Available from: https://seer.cancer.gov/statistics-network/explorer/application.html?site=55&data_type=4&graph_type=5&compareBy=subtype&chk_subtype_622=622&chk_subtype_623=623&chk_subtype_620=620&chk_subtype_621=621&series=age_range&chk_age_range_1=1&chk_age_range_9=9&chk_age_range_141=141&chk_age_range_157=157&sex=3&race=1&stage=106&advopt_precision=1&advopt_show_ci=on&hdn_view=0&advopt_show_apc=on&advopt_display=2#resultsRegion0.

[3] Deluche E, Antoine A, Bachelot T, Lardy-Cleaud A, Dieras V, Brain E, et al. Contemporary outcomes of metastatic breast cancer among 22,000 women from the multicentre ESME cohort 2008–2016. Eur J Cancer. 2020;129:60–70. 10.1016/j.ejca.2020.01.016. 32135312 10.1016/j.ejca.2020.01.016

[4] Side 1 af 20 Om Medicinrådet. 2022. Available from: www.medicinraadet.dk.

[5] Braal CL, Jongbloed EM, Wilting SM, Mathijssen RHJ, Koolen SLW, Jager A. Inhibiting CDK4/6 in Breast Cancer with Palbociclib, Ribociclib, and Abemaciclib: Similarities and Differences. Drugs. 2021;81:317–31. 10.1007/s40265-020-01461-2. 33369721 10.1007/s40265-020-01461-2 PMC7952354

[6] Hanker AB, Sudhan DR, Arteaga CL. Overcoming Endocrine Resistance in Breast Cancer. Cancer Cell. 2020;37:496–513. 10.1016/j.ccell.2020.03.009. 32289273 10.1016/j.ccell.2020.03.009 PMC7169993

[7] MONARCH 3: Interim overall survival (OS) results of abemaciclib plus a nonsteroidal aromatase inhibitor (NSAI) in patients (pts) with HR+, HER2- ad… | OncologyPRO. 2022. Available from: https://oncologypro.esmo.org/meeting-resources/esmo-congress/monarch-3-interim-overall-survival-os-results-of-abemaciclib-plus-a-nonsteroidal-aromatase-inhibitor-nsai-in-patients-pts-with-hr-her2-ad.

[8] Hortobagyi GN, Stemmer SM, Burris HA, Yap YS, Sonke GS, Hart L, et al. Overall Survival with Ribociclib plus Letrozole in Advanced Breast Cancer. N Engl J Med. 2022;386:942–50. 10.1056/NEJMoa2114663. 35263519 10.1056/NEJMoa2114663

[9] Finn RS, Rugo HS, Dieras VC, Harbeck N, Im SA, Gelmon KA, et al. Overall survival (OS) with first-line palbociclib plus letrozole (PAL+LET) versus placebo plus letrozole (PBO+LET) in women with estrogen receptor–positive/human epidermal growth factor receptor 2–negative advanced breast cancer (ER+/HER2− ABC): Analyses from PALOMA-2. J Clin Oncol. 2022;40:LBA1003–LBA1003. 10.1200/JCO20224017_supplLBA1003.

[10] Medicinrådets behandlingsvejledning hæmmere til ER+/ HER2- lokalt fremskreden eller metastatisk brystkræft. 0–123, Medicinrådet 2022. Available from: www.medicinraadet.dk.

[11] Burstein HJ, Somerfield MR, Barton DL, Dorris A, Fallowfield LJ, Jain D, et al. Endocrine Treatment and Targeted Therapy for Hormone Receptor-Positive, Human Epidermal Growth Factor Receptor 2-Negative Metastatic Breast Cancer: ASCO Guideline Update. J Clin Oncol. 2021;39:3959–77. 34324367 10.1200/JCO.21.01392 PMC8659999

[12] Cardoso F, Paluch-Shimon S, Senkus E, Curigliano G, Aapro MS, André F, et al. 5th ESO-ESMO international consensus guidelines for advanced breast cancer (ABC 5). Ann Oncol. 2020;31:1623. 32979513 10.1016/j.annonc.2020.09.010 PMC7510449

[13] Grinshpun A, Tolaney SM, Burstein HJ, Jeselsohn R, Mayer EL. The dilemma of selecting a first line CDK4/6 inhibitor for hormone receptor-positive/HER2-negative metastatic breast cancer. NPJ Breast Cancer. 2023;9:4–7. 36949066 10.1038/s41523-023-00520-7 PMC10033931

[14] Von Elm E, Altman DG, Egger M, Pocock SJ, Gøtzsche PC, Vandenbrouckef JP. The Strengthening the Reporting of Observational Studies in Epidemiology (STROBE) Statement: Guidelines for reporting observational studies. Bull World Health Organ. 2007;85:867–72. 18038077 10.2471/BLT.07.045120 PMC2636253

[15] Schemper M, Smith TL. A note on quantifying follow-up in studies of failure time. Control Clin Trials. 1996;17:343–6. 8889347 10.1016/0197-2456(96)00075-x

[16] Cersosimo RJ. Cyclin-dependent kinase 4/6 inhibitors for the management of advanced or metastatic breast cancer in women. Am J Health Syst Pharm. 2019;76:1183–202. https://pubmed.ncbi.nlm.nih.gov/31369120/. 10.1093/ajhp/zxz121 31369120

[17] Finn RS, Martin M, Rugo HS, Jones S, Im SA, Gelmon K, et al. Palbociclib and Letrozole in Advanced Breast Cancer. N Engl J Med. 2016;375:1925–36. 27959613 10.1056/NEJMoa1607303

[18] Cardoso F, Costa A, Norton L, Senkus E, Aapro M, André F, et al. ESO-ESMO 2nd international consensus guidelines for advanced breast cancer (ABC2). Breast. 2014;23:489–502. 25244983 10.1016/j.breast.2014.08.009

[19] Hortobagyi GN, Stemmer SM, Burris HA, Yap YS, Sonke GS, Paluch-Shimon S, et al. Updated results from MONALEESA-2, a phase III trial of first-line ribociclib plus letrozole versus placebo plus letrozole in hormone receptor-positive, HER2-negative advanced breast cancer. Ann Oncol. 2018;29:1541–7. 29718092 10.1093/annonc/mdy155

[20] Johnston S, Martin M, Di Leo A, Im SA, Awada A, Forrester T, et al. MONARCH 3 final PFS: a randomized study of abemaciclib as initial therapy for advanced breast cancer. NPJ Breast Cancer. 2019;5:1–8. 10.1038/s41523-018-0097-z. 30675515 10.1038/s41523-018-0097-z PMC6336880

[21] Rugo HS, Brufsky A, Liu X, Li B, McRoy L, Chen C, et al. Real-world study of overall survival with palbociclib plus aromatase inhibitor in HR+/HER2− metastatic breast cancer. NPJ Breast Cancer. 2022;8:114. 36220852 10.1038/s41523-022-00479-x PMC9553912

[22] Rugo HS, Liu X, Li B, McRoy L, Chen C, Layman RM, et al. Real-world treatment patterns for palbociclib plus an aromatase inhibitor, or an aromatase inhibitor alone, for patients with metastatic breast cancer in the Flatiron Database. Int J Cancer. 2024;154:701–11. 37831416 10.1002/ijc.34748

[23] West MT, Goodyear SM, Hobbs EA, Kaempf A, Kartika T, Ribkoff J, et al. Real-World Evaluation of Disease Progression After CDK 4/6 Inhibitor Therapy in Patients With Hormone Receptor-Positive Metastatic Breast Cancer. Oncologist. 2023;28:682–90. 36946994 10.1093/oncolo/oyad035 PMC10400146

[24] Saatci O, Huynh-Dam KT, Sahin O. Endocrine resistance in breast cancer: from molecular mechanisms to therapeutic strategies. J Mol Med. 2021;99:1691–710. 34623477 10.1007/s00109-021-02136-5 PMC8611518

[25] Turner NC, Ro J, André F, Loi S, Verma S, Iwata H, et al. Palbociclib in Hormone-Receptor–Positive Advanced Breast Cancer. N Engl J Med. 2015;373:209–19. 26030518 10.1056/NEJMoa1505270

[26] Sledge GW, Toi M, Neven P, Sohn J, Inoue K, Pivot X, et al. MONARCH 2: Abemaciclib in combination with fulvestrant in women with HR+/HER2-advanced breast cancer who had progressed while receiving endocrine therapy. J Clin Oncol. 2017;35:2875–84. 28580882 10.1200/JCO.2017.73.7585

