# Publisher Correction: Real-world effectiveness of CDK 4/6 inhibitors in estrogen-positive metastatic breast cancer

**DOI:** 10.1038/s44276-024-00083-5

**Published:** 2024-09-04

**Authors:** Mathilde Louise Gehrchen, Tobias Berg, Rasmus Garly, Maj-Britt Jensen, Saskia Eßer-Naumann, Jeanette Dupont Rønlev, Hanne Melgaard Nielsen, Ann Knoop, Iben Kümler

**Affiliations:** 1 Danish Breast Cancer Group, Department of Oncology, Copenhagen University Hospital, Rigshospitalet, Copenhagen, Denmark; 2 Department of Oncology, Copenhagen University Hospital, Rigshospitalet, Copenhagen, Denmark; 3 Department of Oncology, Næstved Hospital, Næstved, Denmark; 4 Department of Oncology, University Hospital of Odense, Odense, Denmark; 5 Department of Oncology, Aarhus University Hospital, Aarhus, Denmark; 6 Department of Oncology, Herlev and Gentofte University Hospital, Herlev, Denmark; 7 Department of Clinical Medicine, Faculty of Health and Medical Sciences, University of Copenhagen, Copenhagen, Denmark

Correction to: *BJC Reports* 10.1038/s44276-024-00070-w, published online 20 June 2024

During the production process, the Figs. 2 and 3 have been interchanged.

**Original figures:**

**Figure:**
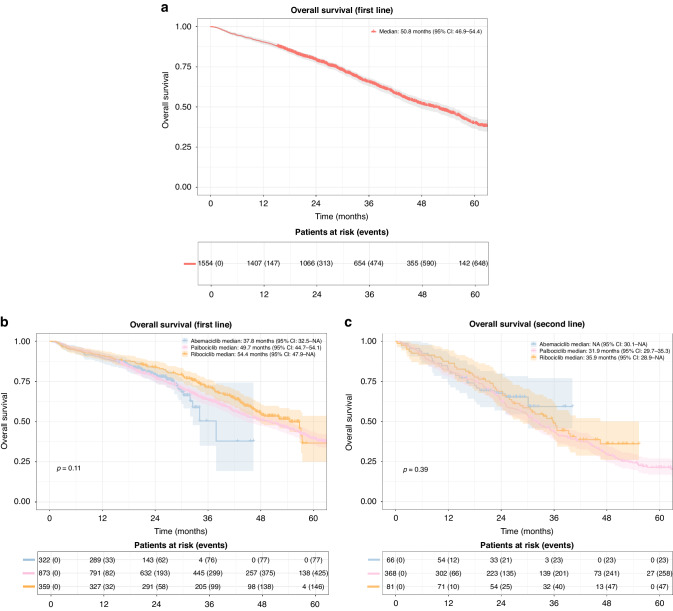
**Fig. 2** **Progression-free survival (PFS)**. **a** Progression-free survival in patients receiving CDK 4/6i treatment in first line. Shaded areas represent confidence intervals. **b** Progression-free survival in patients receiving CDK 4/6i treatment in first line grouped by CDK 4/6i type. Shaded areas represent confidence intervals. **c** Progression-free survival in patients receiving CDK 4/6i treatment in second line grouped by CDK 4/6i type. Shaded areas represent confidence intervals.

**Figure:**
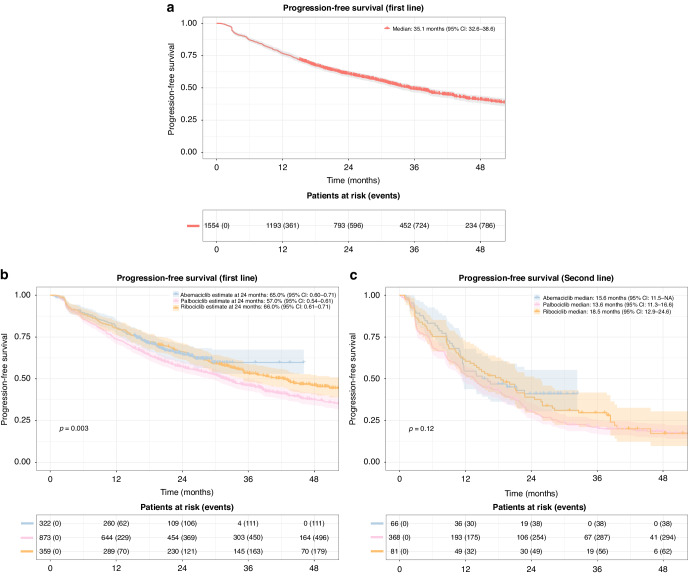
**Fig. 3** **Overall survival (OS)**. **a** Overall survival in patients receiving CDK 4/6i treatment in first line. Shaded areas represent confidence intervals. **b** Overall survival in patients receiving CDK 4/6i treatment in first line grouped by CDK 4/6i type. Shaded areas represent confidence intervals. **c** Overall survival in patients receiving CDK 4/6i treatment in second line grouped by CDK 4/6i type. Shaded areas represent confidence intervals.

**Corrected figures:**

**Figure:**
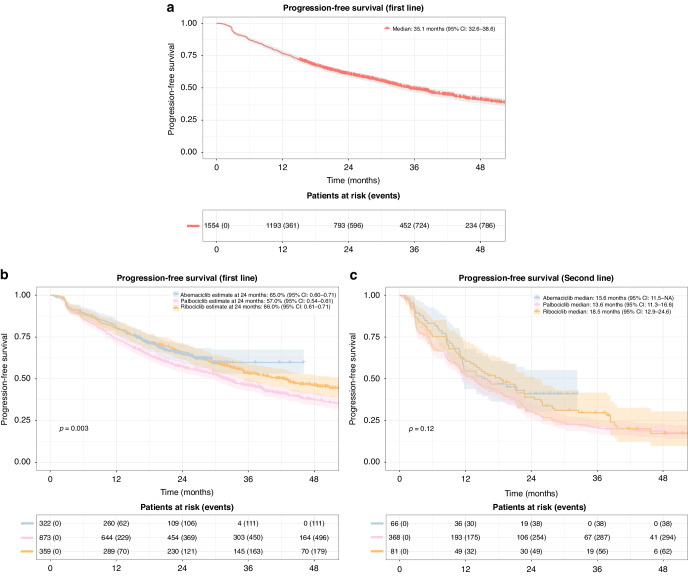
**Fig. 2** **Progression-free survival (PFS)**. **a** Progression-free survival in patients receiving CDK 4/6i treatment in first line. Shaded areas represent confidence intervals. **b** Progression-free survival in patients receiving CDK 4/6i treatment in first line grouped by CDK 4/6i type. Shaded areas represent confidence intervals. **c** Progression-free survival in patients receiving CDK 4/6i treatment in second line grouped by CDK 4/6i type. Shaded areas represent confidence intervals.

**Figure:**
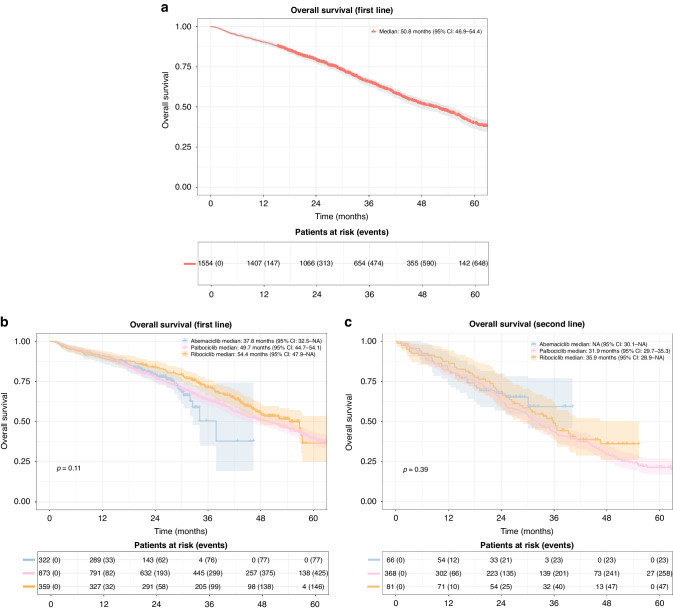
**Fig. 3** **Overall survival (OS)**. **a** Overall survival in patients receiving CDK 4/6i treatment in first line. Shaded areas represent confidence intervals. **b** Overall survival in patients receiving CDK 4/6i treatment in first line grouped by CDK 4/6i type. Shaded areas represent confidence intervals. **c** Overall survival in patients receiving CDK 4/6i treatment in second line grouped by CDK 4/6i type. Shaded areas represent confidence intervals.

The original article has been corrected.

