# Supplementary material for: Real-world effectiveness of CDK 4/6 inhibitors in estrogen-positive metastatic breast cancer
Source: BJC Rep. 2024 Jun 20;2:44. doi: 10.1038/s44276-024-00070-w (PMC11523969; doi:10.1038/s44276-024-00070-w)
Supplement: Supplementary file 1 — Supplementary information [file 44276_2024_70_MOESM1_ESM.docx]

## Supplementary information

**Supplementary Table 1**

Baseline characteristics for patients receiving CDK 4/6i treatment in second line.

|  | **Abemaciclib (n=66)** | **Palbociclib (n=368)** | **Ribociclib (n=81)** | **Overall**  **(n=515)** | **P-value** |
| --- | --- | --- | --- | --- | --- |
| **Age**  Median [range] | 67.2 [40.7, 89.7] | 67.8 [26.6, 89.1] | 70.3 [38.7, 85.5] | 68.2 [26.6, 89.7] | 0.12 |
| **Age**  <40  40-49  50-59  60-69  ≥70 | 0 (0%)  7 (10.6%)  12 (18.2%)  13 (19.7%)  34 (51.5%) | 13 (3.5%)  27 (7.3%)  76 (20.7%)  97 (26.4%)  155 (42.1%) | 1 (1.2%)  7 (8.6%)  10 (12.3%)  22 (27.2%)  41 (50.6%) | 14 (2.7%)  41 (8.0%)  98 (19.0%)  132 (25.6%)  230 (44.7%) | 0.30 |
| **Year of metastatic disease**  2017  2018  2019  2020  2021 | 0 (0%)  0 (0%)  2 (3.0%)  26 (39.4%)  38 (57.6%) | 108 (29.3%)  116 (31.5%)  82 (22.3%)  40 (10.9%)  22 (6.0%) | 1 (1.2%)  20 (24.7%)  31 (38.3%)  27 (33.3%)  2 (2.5%) | 109 (21.2%)  136 (26.4%)  115 (22.3%)  93 (18.1%)  62 (12.0%) | <0.001 |
| **No. of metastatic sites**  1  2  ≥3 | 15 (22.7%)  28 (42.4%)  23 (34.8%) | 80 (21.7%)  115 (31.3%)  173 (47.0%) | 25 (30.9%)  21 (25.9%)  35 (43.2%) | 120 (23.3%)  164 (31.8%)  231 (44.9%) | 0.11 |
| **Sites of metastases**  Non-visceral  Visceral ^a^  Bone-only | 16 (24.2%)  41 (62.1%)  9 (13.6%) | 61 (16.6%)  263 (71.5%)  44 (12.0%) | 14 (17.3%)  51 (63.0%)  16 (19.8%) | 91 (17.7%)  355 (68.9%)  69 (13.4%) | 0.20 |
| **Endocrine backbone**  AI ^b^  Fulvestrant | 26 (39.4%)  40 (60.6%) | 63 (17.1%)  305 (82.9%) | 37 (45.7%)  44 (54.3%) | 126 (24.5%)  389 (75.5%) | <0.001 |
| ^a^ Liver, lungs, CNS, abdominal carcinosis, and/or ovaries.  ^b^ Aromatase inhibitor. | | | | | |

**Supplementary Figure 1**

1. Progression free survival in patients receiving CDK 4/6i treatment in first line based on endocrine sensitivity. The resistant group includes both primary and secondary resistant patients. The shaded areas represent confidence intervals.


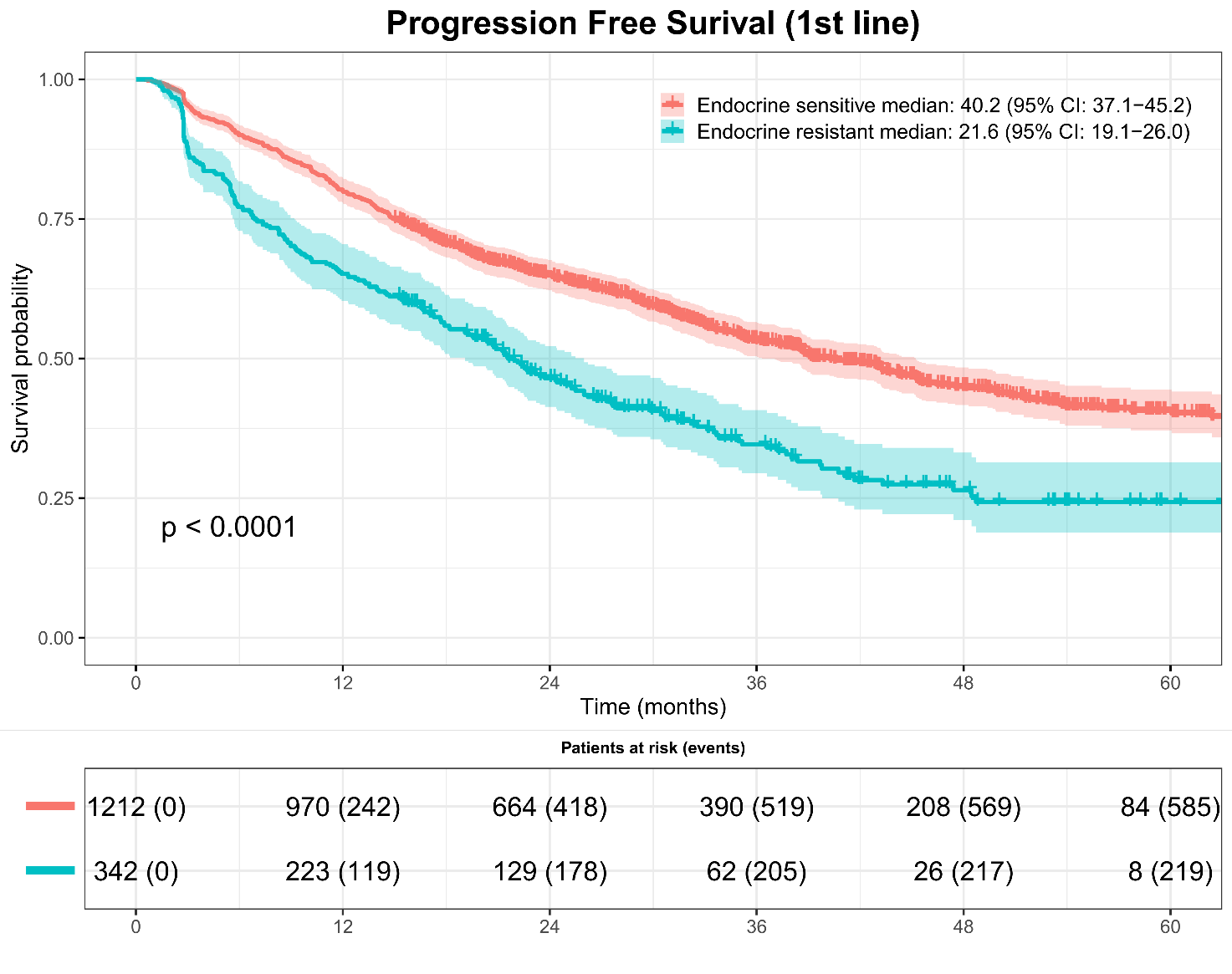


1. Progression free survival in patients receiving CDK 4/6i treatment in second line based on endocrine sensitivity. The resistant group includes both primary and secondary resistant patients. The shaded areas represent confidence intervals.

**
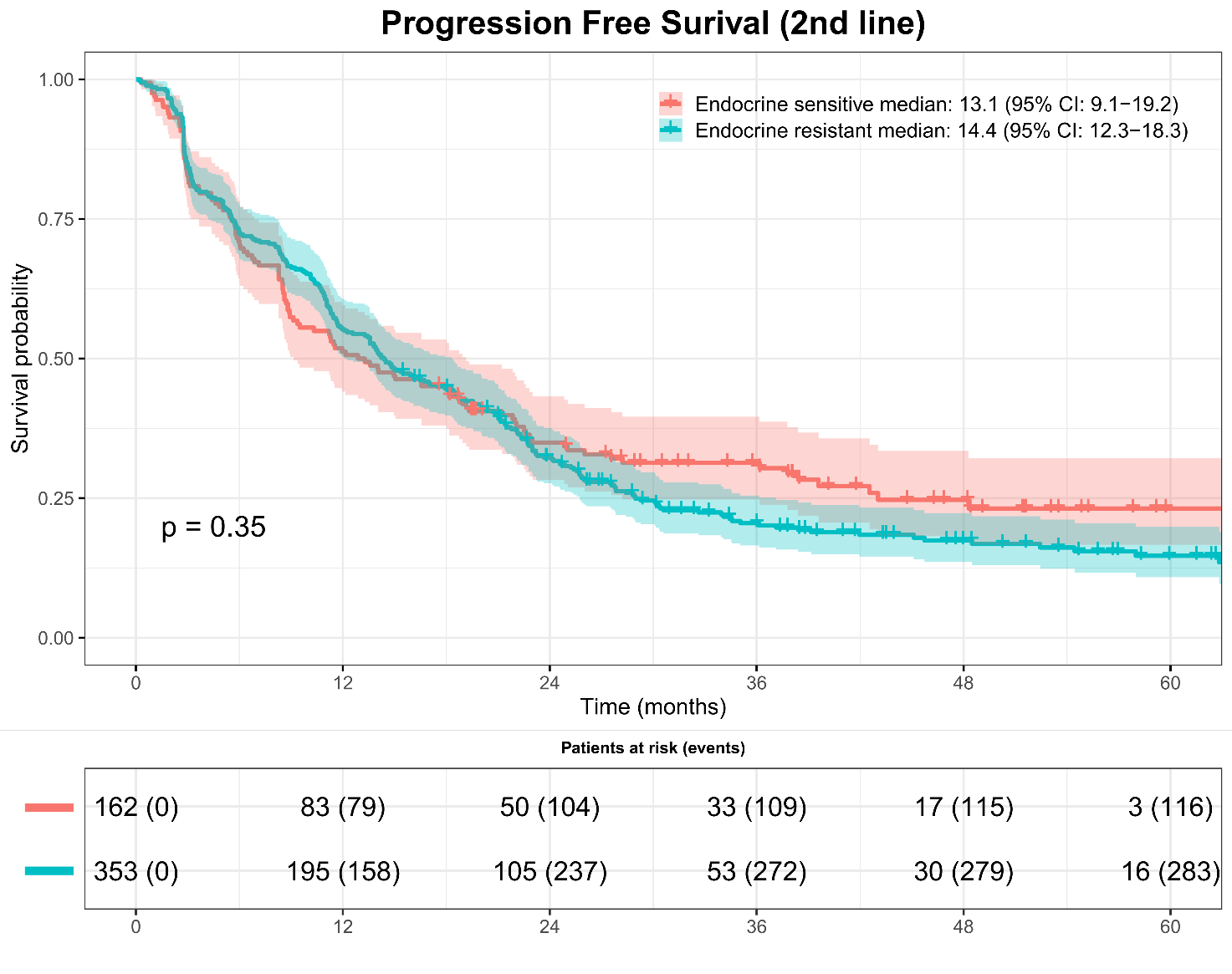
**

**Supplementary Table 2**

Unadjusted and adjusted Cox regression analysis for PFS and OS in patients receiving CDK 4/6i treatment in first line. The adjusted hazard ratios are based on a multivariate model: Cox proportional hazards regression model where PFS is adjusted for age, disease presentation, endocrine backbone, and endocrine sensitivity.

|  | **Hazard ratio (95% CI ^a^)** | | | |
| --- | --- | --- | --- | --- |
|  | Unadjusted | | Adjusted | |
|  | HR (95% CI) | p-value | HR (95% CI) | p-value |
| **CDK 4/6i^b^** | | | | |
| Palbociclib  Abemaciclib  Ribociclib | Ref. ^c^  0.77 (0.62-0.94)  0.78 (0.66-0.93) | Ref.  0.01*  0.004* | Ref.  0.74 (0.60-0.90)  0.80 (0.68-0.96) | Ref.  0.005  0.01 |
| **Age** | | | | |
|  | 0.99 (0.98-0.99) | < 0.001* | 0.99 (0.98-0.99) | < 0.001 |
| **Sites of metastases** | | | | |
| Non-visceral  Visceral  Bone-only | Ref.  1.20 (1.00-1.44)  0.91 (0.74-1.13) | Ref.  0.047*  0.39 | Ref.  1.09 (0.91-1.32)  0.80 (0.65-0.9992) | Ref.  0.34  0.049 |
| **Endocrine backbone** | | | | |
| Letrozole  Anastrozole  Exemestane  Fulvestrant | Ref.  1.95 (0.93-4.12)  1.12 (0.76-1.65)  1.46 (1.26-1.69) | Ref.  0.08  0.57  < 0.001* | Ref.  2.12 (1.01-4.48)  1.24 (0.84-1.83)  1.30 (1.11-1.53) | Ref.  0.048  0.28  < 0.001 |
| **Endocrine sensitivity** | | | | |
| Sensitive  Primary resistant  Secondary resistant | Ref.  1.88 (1.45-2.44)  1.67 (1.40-2.00) | Ref.  < 0.001*  < 0.001* | Ref.  1.69 (1.28-2.22)  1.44 (1.18-1.76) | Ref.  < 0.001  < 0.001 |
| ^a^ Confidence interval  ^b^ Cycline dependent kinase 4/6 inhibitor  ^c^ Reference | | | | |

**Supplementary Figure 2**

1. Sankey plot for n = 172 patients changing CDK 4/6i within first line treatment.


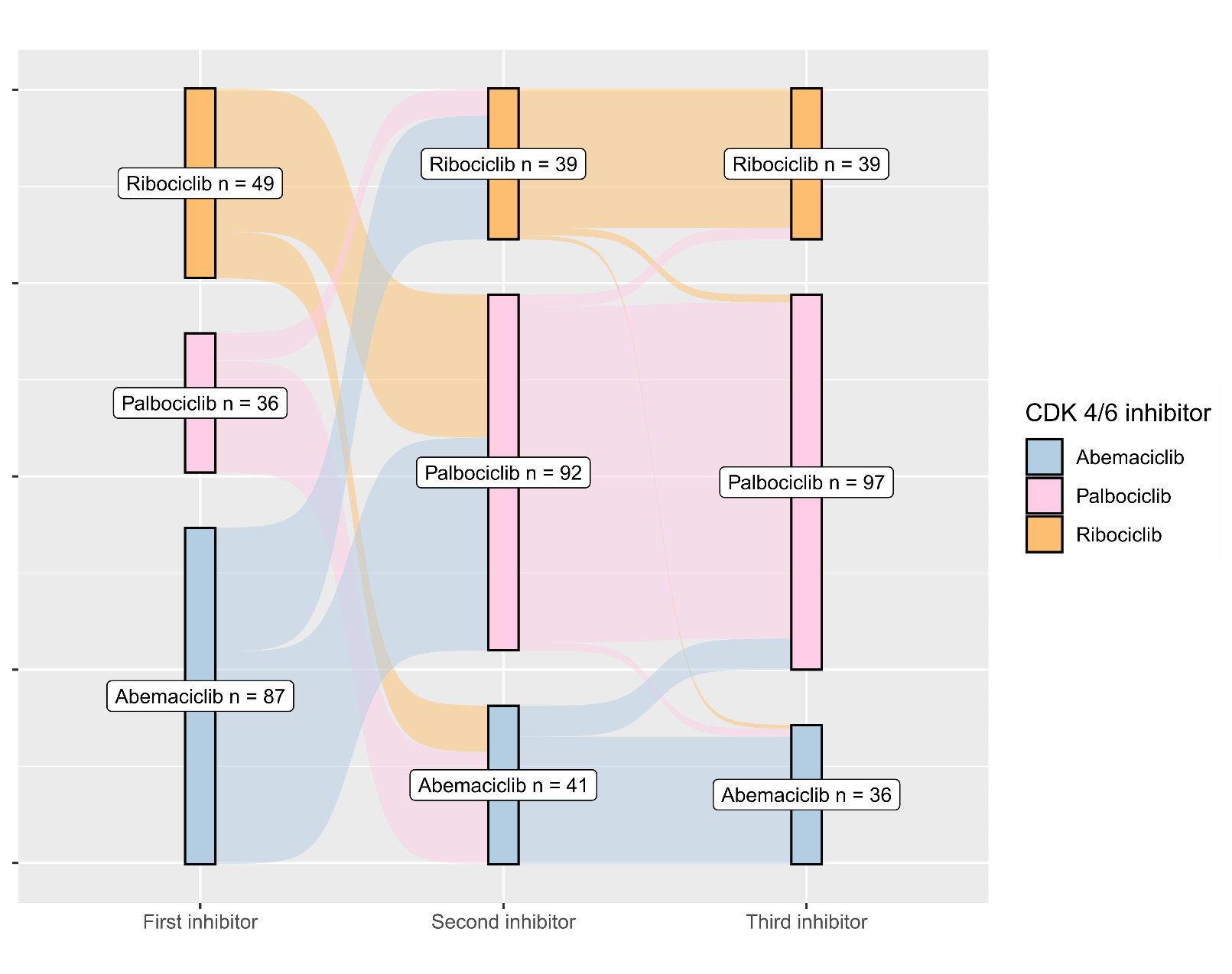


1. Sankey plot for n = 37 patients changing CDK 4/6i within second line treatment.


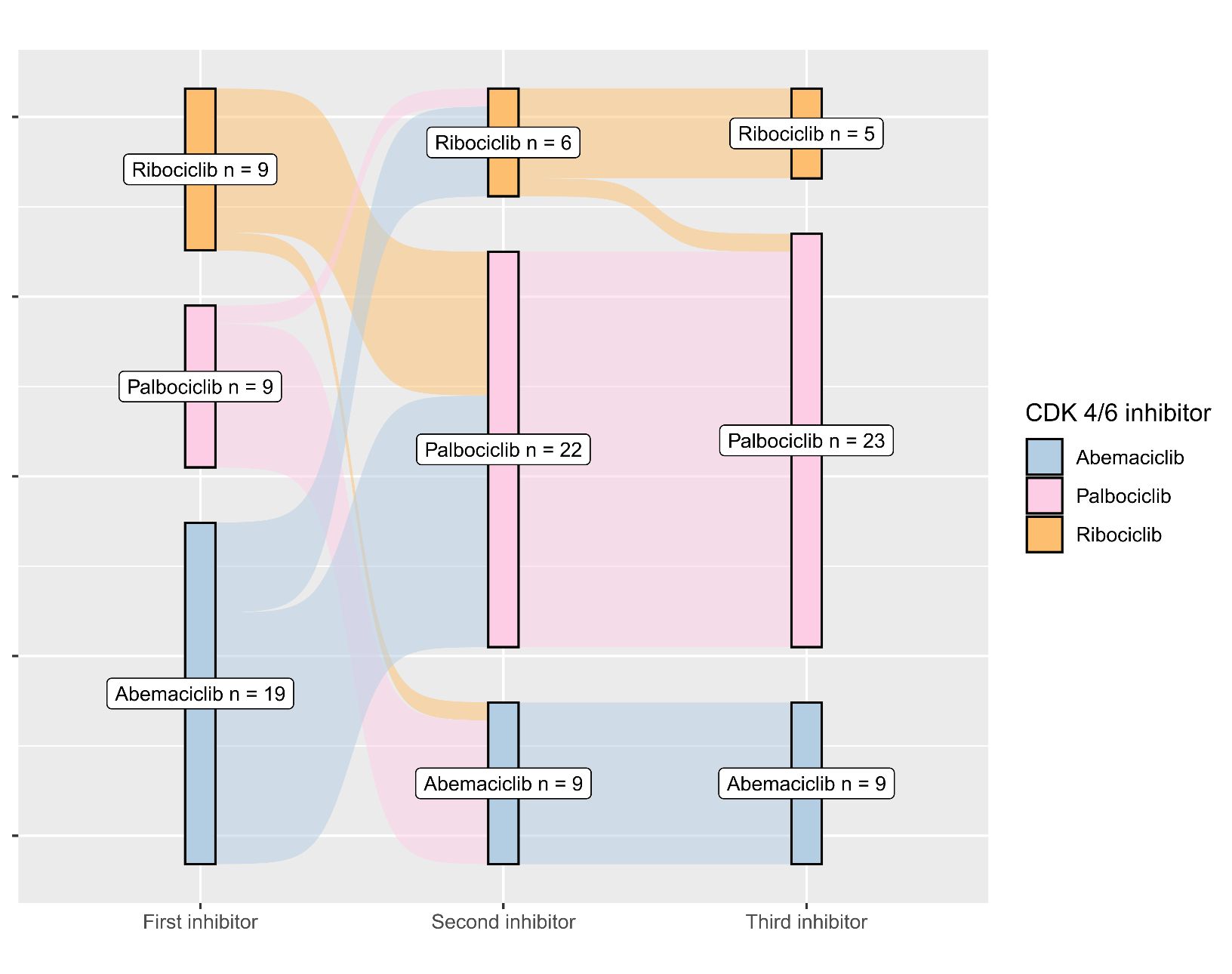


**Supplementary Table 3**

Reasons for discontinuation of initial CDK 4/6i treatment.

|  | **Abemaciclib**  **(n = 388)** | **Palbociclib**  **(n = 1241)** | **Ribociclib**  **(n = 440)** |
| --- | --- | --- | --- |
| **Reason for discontinuation** | | | |
| Toxicities  Progression  Death  Other reason ^a^ | 164 (42.3%)  80 (20.6%)  5 (1.3%)  23 (5.9%) | 187 (15.1%)  624 (50.3%)  22 (1.8%)  156 (12.6%) | 97 (22.0%)  178 (40.5%)  8 (1.8%)  54 (12.3%) |
| **Patients still on treatment** | | | |
|  | 116 (29.9%) | 252 (20.3%) | 103 (23.4%) |
| ^a^ Other reasons for treatment discontinuation includes e.g. decreased general condition, treatment for other disease or malignancy, patient’s own wish, and pause in the CDK 4/6i treatment. | | | |

**Supplementary Table 4**

1. Endocrine sensitivity in first line of treatment. The p-values are based on a chi square test comparing the three CDK 4/6i groups receiving aromatase inhibitor (AI) and fulvestrant (F), respectively.

|  | **Abemaciclib** | | **Palbociclib** | | **Ribociclib** | | **P-value** |
| --- | --- | --- | --- | --- | --- | --- | --- |
| **Endocrine backbone** | AI  (n = 255) | Fulvestrant (n=67) | AI  (n=580) | Fulvestrant (n=293) | AI  (n=265) | Fulvestrant (n=94) | AI:  0.08  F:  0.02 |
| **Endocrine sensitivity** |  |  |  |  |  |  |  |
| Sensitive ^a^  Primary resistant ^b^  Secondary resistant ^c^ | 215 (84.3%)  15 (5.9%)  25 (9.8%) | 27 (40.3%)  8 (11.9%)  32 (47.8%) | 513 (88.4%)  25 (4.3%)  42 (7.2%) | 177 (60.4%)  34 (11.6%)  82 (28.0%) | 229 (86.4%)  6 (2.3%)  30 (11.3%) | 51 (54.3%)  9 (9.6%)  34 (36.2%) |  |
| ^a^ Patients who had not received previous endocrine therapy, patients with primary metastatic breast cancer, patients who had recurrence ≥ 12 months after ending adjuvant endocrine therapy.  ^b^ Patients who had recurrence during the first 2 years of adjuvant endocrine therapy, while still being on endocrine therapy.  ^c^ Patients who had recurrence after the first 2 years of adjuvant endocrine therapy, while still being on endocrine therapy, or recurrence occurred within 12 months of completing adjuvant endocrine therapy. | | | | | | | |

1. Endocrine sensitivity in second line of treatment. The p-values are based on Fischer’s Exact Test comparing the three CDK 4/6i groups receiving aromatase inhibitor (AI) and fulvestrant (F), respectively.

|  | **Abemaciclib** | | **Palbociclib** | | **Ribociclib** | | **P-value** |
| --- | --- | --- | --- | --- | --- | --- | --- |
| **Endocrine backbone** | AI  (n = 26) | Fulvestrant  (n = 40) | AI  (n = 63) | Fulvestrant  (n = 305) | AI  (n = 37) | Fulvestrant  (n = 44) | AI:  0.59  F:  0.84 |
| **Endocrine sensitivity** |  |  |  |  |  |  |  |
| Sensitive ^a^  Primary resistant ^b^  Secondary resistant ^c^ | 15 (57.7%)  0 (0%)  11 (42.3%) | 13 (32.5%)  2 (5.0%)  25 (62.5%) | 29 (46.0%)  6 (9.5%)  28 (44.4%) | 74 (24.3%)  19 (6.2%)  212 (69.5%) | 19 (51.4%)  2 (5.4%)  16 (43.2%) | 12 (27.3%)  2 (4.5%)  30 (68.2%) |  |
| ^a^ Patients who did not receive endocrine therapy in first line treatment or patients who progressed ≥ 1 month after ending endocrine therapy.  ^b^ Patients who progressed during the first 6 months of endocrine therapy in first line treatment, while still being on or within 1 month of ending endocrine therapy.  ^c^ Patients who progressed ≥ 6 months after start of endocrine therapy in first line treatment, while still being on or within 1 month of ending endocrine therapy. | | | | | | | |
